# Characterization of Novel Progression Factors in Castration-Resistant Prostate Cancer Based on Global Comparative Proteome Analysis

**DOI:** 10.3390/cancers13143432

**Published:** 2021-07-08

**Authors:** Ann-Yae Na, Soyoung Choi, Eunju Yang, Kwang-Hyeon Liu, Sunghwan Kim, Hyun Jin Jung, Youngshik Choe, Yun-Sok Ha, Tae Gyun Kwon, Jun Nyung Lee, Sangkyu Lee

**Affiliations:** 1 BK21 FOUR Community-Based Intelligent Novel Drug Discovery Education Unit, College of Pharmacy, Kyungpook National University, Daegu 41566, Korea; cpblady@hanmail.net (A.-Y.N.); sylvdh@knu.ac.kr (S.C.); dstlkh@knu.ac.kr (K.-H.L.); 2 Research Institute of Pharmaceutical Sciences, Kyungpook National University, Daegu 41566, Korea; ejy125@gmail.com; 3 Mass Spectrometry Convergence Research Center and Green-Nano Materials Research Center, Daegu 41566, Korea; sunghwank@knu.ac.kr; 4 Department of Chemistry, Kyungpook National University, Daegu 41566, Korea; 5 Korea Brain Research Institute, Daegu 41068, Korea; junghj@kbri.re.kr (H.J.J.); dallarae@gmail.com (Y.C.); 6 Department of Urology, School of Medicine, Kyungpook National University, Daegu 41405, Korea; yunsokha@gmail.com (Y.-S.H.); tgkwon@knu.ac.kr (T.G.K.); 7 Joint Institute for Regenerative Medicine, Kyungpook National University, Daegu 41405, Korea

**Keywords:** prostate cancer, androgen deprivation therapy, comparative proteomics, castration-resistant prostate cancer, FOXA1, HMGN

## Abstract

**Simple Summary:**

Here, we investigated prostate cancer (PCa) tissues at each stage of progression, from benign prostatic hyperplasia to castration-resistant prostate cancer (CRPC), based on quantitative proteomic technology, including tissues after androgen deprivation therapy (ADT). In total, we identified 4768 proteins, and 4069 of them were quantified. We performed a systematic bioinformatics analysis of 865 differentially expressed proteins (DEPs) in the combined PCa tissues. We found 15 DEPs, including FOXA1 and HMGN1–3, as novel factors were significantly involved in the progression to CRPC after ADT in T3G3. All targets were verified to have increased levels of FOXA1 and HMGN1–3 in CRPC by immunoblotting and indirect enzyme-linked immunosorbent assay. The FOXA1 and HMGN1–3 proteins could be used as CRPC-related factors in clinical therapeutic agents.

**Abstract:**

Identifying the biological change from hormone-naïve prostate cancer to castration-resistant prostate cancer (CRPC) is a major clinical challenge for developing therapeutic agents. Although the pathways that lead to CRPC are not fully completely understood, recent evidence demonstrates that androgen signaling is often maintained through varied mechanisms. Androgen deprivation therapy (ADT) is used as a primary treatment for preventing the progression of prostate cancer (PCa). Here we investigated PCa tissues at each stage of progression, from benign prostatic hyperplasia (BPH) to CRPC, based on quantitative proteomic technology, including tissues after ADT. In total, 4768 proteins were identified in this study, of which 4069 were quantified in the combined PCa tissues. Among the quantified proteins, 865 were differentially expressed proteins (21.2%). Based on the quantitative protein results, we performed systematic bioinformatics analysis and found that the levels of 15 proteins, including FOXA1 and HMGN1–3, increased among T3G3, T3GX, and CRPC, despite the ADT. Among all targets, we verified the increased levels of FOXA1 and HMGN1–3 in CRPC by immunoblotting and indirect enzyme-linked immunosorbent assay. In summary, we discuss the changes in intracellular factors involved in the progression of CRPC PCa despite ADT. Moreover, we suggest that FOXA1 and HMGN1–3 proteins could be used as potential CRPC-related factors in clinical therapeutic agents.

## 1. Introduction

Prostate cancer (PCa) is one of the most diagnosed cancers in men. Although localized PCa is highly curable owing to advances in screening and early detection, approximately 20% of patients with PCa continue to exhibit advanced or metastatic cancers [1]. Androgen deprivation therapy (ADT) is the standard care for patients with PCa after definitive primary therapy, and it provides an initial benefit and rapid therapy response [2]. Unfortunately, nearly all patients eventually develop resistance against ADT, resulting in castration-resistant prostate cancer (CRPC). When PCa progresses to CRPC, the prognosis rapidly deteriorates. For example, men with nonmetastatic CRPC have a worse prognosis and are notably at a higher risk of developing bone metastasis than their hormone-sensitive counterparts [3]. Among nonmetastatic CRPC men, approximately 60% developed metastatic disease during the first 5 years, with most of the metastasis occurring within the first 3 years [4].

In the major bottlenecks for overcoming PCa, identifying the biological mechanism of conversion from hormone-naïve PCa to CRPC is the most clinically important to develop therapeutic agents. Although the pathways that lead to CRPC are not fully understood, recent evidence demonstrates that androgen signaling is often maintained through varied mechanisms [5]. The majority of mechanisms that are identified to lead to CRPC are mediated by androgen receptor (AR)-dependent pathways, including AR gene amplification and protein overexpression, mutation, hypersensitivity pathway, and variants [6,7]. Moreover, other diverse mechanisms are reported in the literature, such as regulation of intratumoral androgen biosynthesis, progesterone and glucocorticoid receptor activation, reciprocal regulation of PI3K/AKT/mTOR pathway, and coactivator overexpression [5,6]. Castration resistance may simultaneously involve several of these processes; however, the mechanism involved in the progression to CRPC despite ADT is not yet known.

Proteomics, a large-scale study of protein, can provide comprehensive and quantitative information on changes in proteins representing the biological mechanisms of PCa progress. There are several studies that identified diagnostic biomarkers for PCa based on comparative proteomic research techniques instead of prostate-specific antigen (PSA) [8,9]. Because the direct protein profiling of the tissues of patients with PCa at each stage of progression provides information to understand the detailed molecular mechanisms involved in cancer progression, various studies were conducted [10,11,12]. However, few comparative quantitative proteome research findings in tissues were obtained from patients before and after ADT among previous studies. Because most cases of CRPC occur after ADT therapy, proteomic changes in PCa tissues before and after ADT can provide information on intracellular mechanical changes. As most of the patients in whom the ADT fails progress to CRPC, the survival rate rapidly decreases. Therefore, it is necessary to investigate the progression mechanism to CRPC after ADT. Here, we analyzed PCa tissues at each stage of progression, from benign prostatic hyperplasia (BPH) to CRPC based on quantitative proteomic technology, including tissues after ADT therapy. We quantified 4069 proteins and identified those with significantly changed expression levels after ADT through bioinformatics analysis. With such data, we suggest the proteins that could be responsible for CRPC-related factors.

## 2. Materials and Methods

### 2.1. Clinical Tissues of Patients with Prostate Cancer

The biospecimens and data used for this study were provided by the National Biobank of Korea-Kyungpook National University Hospital, a member of the Korea Biobank Network-KNUH, and were obtained (with informed consent) under Institutional Review Board-approved protocols (approval number: KNUMC 2016-05-021). All samples were obtained from patients treated at the Kyungpook National University Chilgok Hospital. All tumor samples were acquired after obtaining patient consent for tissue sample donation and after an examination. The diagnosis of PCa was verified based on the outcomes of pathological analyses after surgery. All PCa tissues used for proteomics were collected from the primary lesion of the prostate. The CRPC tissues were obtained during transurethral resection of prostate in patients with CRPC. To prevent contamination from the interstitial benign lesion, we used the resected prostate tissues from the sites with obvious progression on the sequential image study, such as computed tomography or magnetic resonance imaging. The other PCa tissues (T2G2, T3G2, T3G3, and T3GX) were obtained after radical prostatectomy in patients with hormone sensitive PCa. After performing radical prostatectomy, a palpable mass was identified in the extracted prostate, and cancer tissues were collected by incising the main mass. The main mass from which the tissues were extracted was later subjected to pathological examination to confirm information on the pathological stage and grade of the tumor. In this study, we only used the prostatic adenocarcinoma tissue as histopathological type diagnosed on post-operative pathologic examination. All tissues used in this study were collected from different cases. Both the normal tissues and PCa tissues were stored at −80 °C before use. According to tumor type and Gleason score, the PCa tissues used were classified into T2G2, T3G2, and T3G3. T3GX refers to stable PCa during ADT. T and G represent the pathological tumor stage as per the American Joint Committee on Cancer TNM Staging System for Prostate Cancer (8th edition, 2017) and the grade group (1–5) as per the 2014 International Society of Urological Pathology Consensus Conference determined at the final histological examination after surgery, respectively [13].

### 2.2. Sample Preparation for Comparative Proteome Analysis

Three PCa tissues (20 µg) in each group were homogenized, and proteins were extracted with RIPA buffer (Thermo Fisher Scientific, Waltham, MA, USA) containing the Halt protease inhibitor cocktail (Thermo Fisher Scientific, Waltham, MA, USA). The samples were centrifuged at 12,000× *g* for 10 min at 4 °C. Five hundred micrograms of protein were pooled from each group sample and were prepared after protein quantification using the bicinchoninic acid (BCA) colorimetric assay kit (Thermo Fisher Scientific, Waltham, MA, USA). In the protein lysate, 15 mM dithiothreitol was added for reduction, followed by incubation at 56 °C for 30 min and addition of 60 mM iodoacetamide for alkylation at RT for 45 min in the dark. The protein lysate purification was started with the addition of 10% trichloroacetic acid at −20 °C for 4 h and then was washed twice with ice-cold acetone. The solution was centrifuged at 16,000× *g* for 10 min, and the pellet was suspended in 100 mM ammonium bicarbonate buffer. Next, trypsin/P (Thermo Fisher Scientific, Rockford, IL, USA) was added to digest protein with an enzyme-to-protein ratio of 1:50 (*w*/*w*) and incubated at 37 °C overnight. The quenching step was performed with 1% Trifluoroacetic acid (TFA) (*v*/*v*) in a digested peptide, with centrifugation at 16,000× *g* at 4 °C for 10 min. The purified peptide concentration was measured using a quantitative, colorimetric peptide assay kit (Thermo Fisher Scientific, Waltham, MA, USA).

The dried peptides were resuspended in 100 mM triethylammonium bicarbonate and equivalent 80-µg peptides were prepared in each group for 6-plex tandem mass tags (TMT) labeling (Thermo Fisher Scientific, Waltham, MA, USA). A TMT label reagent was dissolved in 41 µL of acetonitrile (ACN) and incubated for 1 h at room temperature. The BPH, T2G2, T3G2, T3G3, T3GX, and CRPC groups were 6-plex TMT labeled 126–131. The TMT labeling reaction was quenched with the addition of 8 µL of 5% hydroxylamine into each sample and incubation for 15 min at room temperature. Equal sample amounts were combined in a Lobind^®^ e-tube (Eppendorf, Hamburg, Germany) and speed-vacuum dried.

### 2.3. Peptide Fractionation by High-pH Reverse-Phase and Desalting

Peptide fractionation (100 µg) was performed using a high-pH reverse-phase fractionation kit (Thermo Fisher Scientific, Rockford, IL, USA). Eight different pH values in 0.1% triethylamine with 10%, 12.5%, 15%, 17.5%, 20%, 22.5%, 25%, and 50% ACN were used to elute fractionated peptides. A total of eight fractionated peptide samples were desalted with ZipTip C18 resin (Millipore, Burlington, MA, USA) and dried again for analysis with liquid chromatography–tandem mass spectrometry (LC–MS/MS).

### 2.4. Nano-Liquid Chromatography–Tandem Mass Spectrometry Analysis

For accurate mass measurement, the LTQ Velos-Orbitrap Mass Spectrometer (Thermo Fisher Scientific) connected to an Eksigent nano-liquid chromatograph (Thermo Fisher Scientific) and a Q-Exactive Plus Orbitrap (Thermo Fisher) connected with nano-liquid chromatography system (Dionex) were used. The LTQ Velos-Orbitrap LC–MS/MS at Mass Spectrometry Convergence Research Center were supported. The condition LC gradient method was run a total of 75-min gradient method with a linear gradient of 5–30% solvent B (ACN/0.1% formic acid) at a constant flow rate of 300 nL/min. The injection volume was 2 µL of 500 ng peptide dissolved in solvent A (water/0.1% formic acid). Peptide separation was performed using a homemade C12 column (0.75 mm i.d., 10 cm; Phenomenex Inc., Torrance, CA, USA) filled with Jupiter C12 resin, which was used as the analytical column. For a separate peptide, we used a linear gradient of 0–21% solution B (100% ACN in 0.1% FA) for 110 min, 21–90% solvent B for 8 min, and 90% solvent B for 10 min at a sustained flow rate of 300 nL/min. The eluted peptides were sprayed into the Orbitrap mass spectrometer at 1.8 kV. A full scan from *m*/*z* 300–1800 at a resolution of 30,000 was examined. The top 10 modes followed by a data-dependent acquisition of the MS/MS scan method was applied using high-energy collision dissociation with a normalized collision energy of 40 eV. Single charged peptides were excluded from fragmentation. The analysis was performed using an LTQ Velos-Orbitrap supported by Basic Science Research Capacity Enhancement Project through Korea Basic Science Institute (National Research Facilities and Equipment Center). The same batch of eluted peptides was loaded onto a Q-Exactive Orbitrap spectrometer and separated on an Ultimate 3000 RSLCnano System with a PepMap 100 C18 LC column (#164535) as a loading column followed by PepMap RSLC C18 (#ES803) analytical column at a flow rate of 300 nL/min for 135 min. The desalted peptide mixtures ware fractioned in a linear gradient for 0–5% solvent B for 10 min, 5–35% solvent B for 60 min, 80% solvent B for 12 min, and 5% solvent B for 25 min. The nano and trap column temperatures were set at 45 °C. Data were collected over a broad mass to charge (*m*/*z*) precursor full mass selection scan range of *m*/*a* 350–2000. A full scan MS with a data-dependent MS/MS acquisition was performed in a range of 350–2000 *m*/*z*. The mass spectrometry proteomics data have been deposited into the ProteomeXchange Consortium via the PRIDE partner repository with the dataset identifier PXD023592.

### 2.5. Peak Alignment and Database Search

Peptides and proteins were identified using the MaxQuant search engine (v.1.5.2.8) against the Uniprot human database (released in 2018, 73,928 sequences; 25,105,724 residues) with the following search parameters: reverse decoy database, trypsin/P specificity, up to two missing cleavages sites, and a precursor mass tolerance of 20 ppm. The fragment ion mass to tolerance was set at 0.02 Da. Carbamidomethyl addition on Cys residues (+57.02146 Da) was a fixed modification; acetylation on protein N-terminal (+42.0105 Da) and oxidation on Met residues (+15.9949 Da) were variable modifications. The false discovery rate (FDR) was set to 0.01, and the minimum score for peptides was adjusted to be at least over 40.

### 2.6. Bioinformatics Analysis

A bioinformatics study was performed to analyze differentially expressed proteins (DEPs) using the UniProt-GOA database (http://www.ebi.ac.uk/GOA/ (accessed on 13 December 2018)) and DAVID (https://david.ncifcrf.gov/ (accessed on 13 November 2020)). For gene ontology (GO) annotation, log2 values of proteins with over two-fold change were classified based on three categories: biological process, cellular component, and molecular function. A pathway analysis online service, Kyoto Encyclopedia of Genes and Genomes (KEGG) mapper (https://www.genome.jp/kegg/mapper.html (accessed on 1 October 2020)), was used for mapping the annotation results. The DEPs were also transformed for z-scores and then clustered by one-way hierarchical clustering in the Perseus software platform (v. 1.6) into seven classification types. The cluster memberships were visualized with a heat map using the “heatmap.2” function from the “gplots” R-package. Web-free Software of STRING network (https://string-db.org/ (accessed on 17 October 2020)) was used based on the highest confidence (0.900) of required interaction score.

### 2.7. Western Blotting

Tissue sample (100 µg) was added to the RIPA buffer (Thermo, Waltham, MA, USA) for homogenization and then subjected to centrifugation to obtain the supernatant. Protein concentration was quantified using the BCA assay kit (Thermo, Waltham, Massachusetts). The tissue lysates were separated using 10% SDS–PAGE gel for 90 min and transferred to polyvinylidenefluoride membranes (Roche, Basel, Switzerland) for 2 h. After blocking with 5% BSA in Tris-buffered saline for 1 h. Then, the blots were incubated with primary antibodies for FoxA1 (Cell signaling, Beverly, MA, USA), HMGN1 (Cell signaling, Beverly, MA, USA), HMGN2 (Cell signaling, Beverly, MA, USA), HMGN3 (Abcam, Cambridge, UK), and TSBP1 (Abcam, Cambridge, UK) overnight, after which they were reacted with an HRP-conjugated secondary antibody for 2 h. An enhanced chemiluminescence prime western blotting detection reagent (GE Healthcare, Chalfont St. Giles, UK) was used to visualize the protein bands. The density of respective bands was analyzed with the iBright 1500 (Thermo, Waltham, MA, USA).

### 2.8. Indirect Enzyme-Linked Immunosorbent Assay

One µg of tissue lysate sample was mixed with 50 µL of coating buffer (0.2 M Sodium bicarbonate pH 9.4) and incubated overnight at 4 °C in a 96-well plate (Corning, NY, USA). Next, it was washed three times with wash buffer (TBS containing 0.05% Tween 20, pH 7.2) and then incubated for 7 h at room temperature in the blocking buffer (3% BSA in TBS-T). The primary antibodies against FOXA1 (cell signaling), HMGN1 (cell signaling), HMGN2 (cell signaling), HMGN3 (Abcam), and TSBP1 (Abcam) were added to each well, followed by overnight incubation at 4 °C. After washing plate three times with TBS, HRP-conjugated rabbit antibody was added to each well and incubated for 3 h. Finally, after washing three times with TBS, and the TMB substrate solution (Thermo Fisher Scientific, Waltham, MA, USA) was added to each well. After 5–25 min, when the color changed appositely, a stop solution was added to each well. The absorbance of the plate was measured using a microplate reader (TECAN, Switzerland) at 450 nm.

## 3. Results

### 3.1. Comparative Global Protein Profiling in Prostate Cancer Tissues

**Figure 1:**
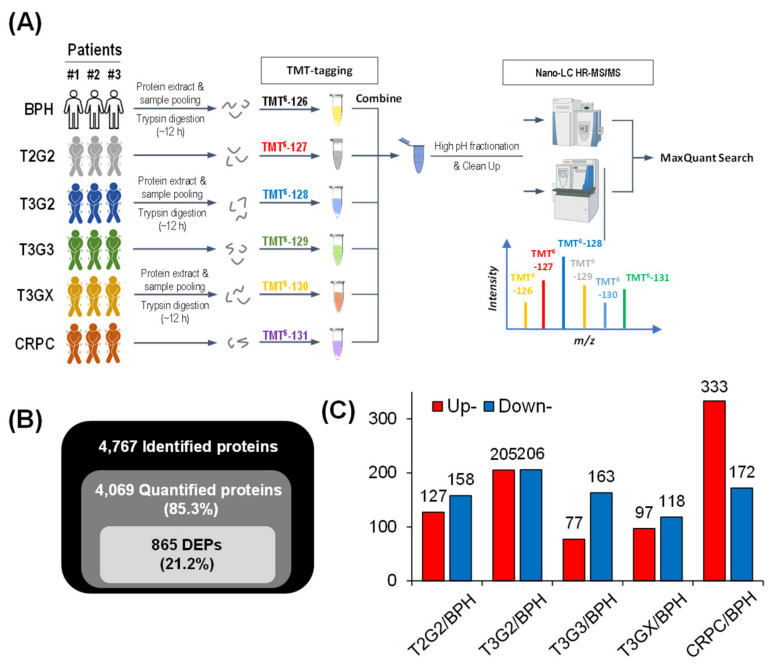
A bottom-up proteomic technique using nano-liquid chromatography–tandem mass spectrometry in six groups of different patients with prostate cancer. (**A**) Proteomic experimental scheme of 6-plex tandem mass tags labeled prostate tissue. (**B**) The basic statistical figure of mass spectrometry results. (**C**) Histogram of the distribution of differentially expressed proteins in different comparison groups.

To identify protein factors involved in the progression of CRPC, the MS-based quantitative proteomics approach based on 6-plex TMT was performed using the patient tissues from T2G2 to CRPC and BPH patient tissues were used as a control. The PCa tissues used in the analysis comprised tissues for each progression stage from T2G2 to T3G3, T3GX obtained after ADT, and CRPC. The experimental workflow is depicted in Figure 1A. PCa tissues of three patients at each stage were pooled for homogenization. The pooled samples were subjected to trypsin digestion, and TMT-tagging was independently performed twice. Each prepared sample was quantitatively analyzed in two types of high resolution and accuracy mass spectrometers, such as LTQ Velos-Orbitrap and Q-Exactive mass spectrometer. The MS/MS spectra produced were assessed using MaxQuant 1.5 to identify and quantify the peptides and proteins involved, allowing a maximum FDR of 1% for the proteins and peptides after excluding contamination (Table S1).

In this study, a total of 415,347 MS/MS spectra were obtained with MS. The analysis results showed that 118,414 (28.5%) spectra matched with 54,271 peptides, among which 51,980 were unique. In total, 4768 proteins were identified in this study, among which 4069 were quantified in the combined PCa tissues. The proteins were classified into two categories based on their relative levels. Proteins with a quantitative ratio of >2.0 were considered upregulated and those with a quantitative ratio of <0.5 were deemed downregulated. Among the quantified proteins, there were 865 DEPs (21.2%; Figure 1B,C). We implemented a quality check for the proteins and results from the MS-based proteomics with the TMT labeling using scatter plots and bioinformatics analysis, respectively (Figure S1).

### 3.2. Bioinformatics Analysis of Prostate Tissues

**Figure 2:**
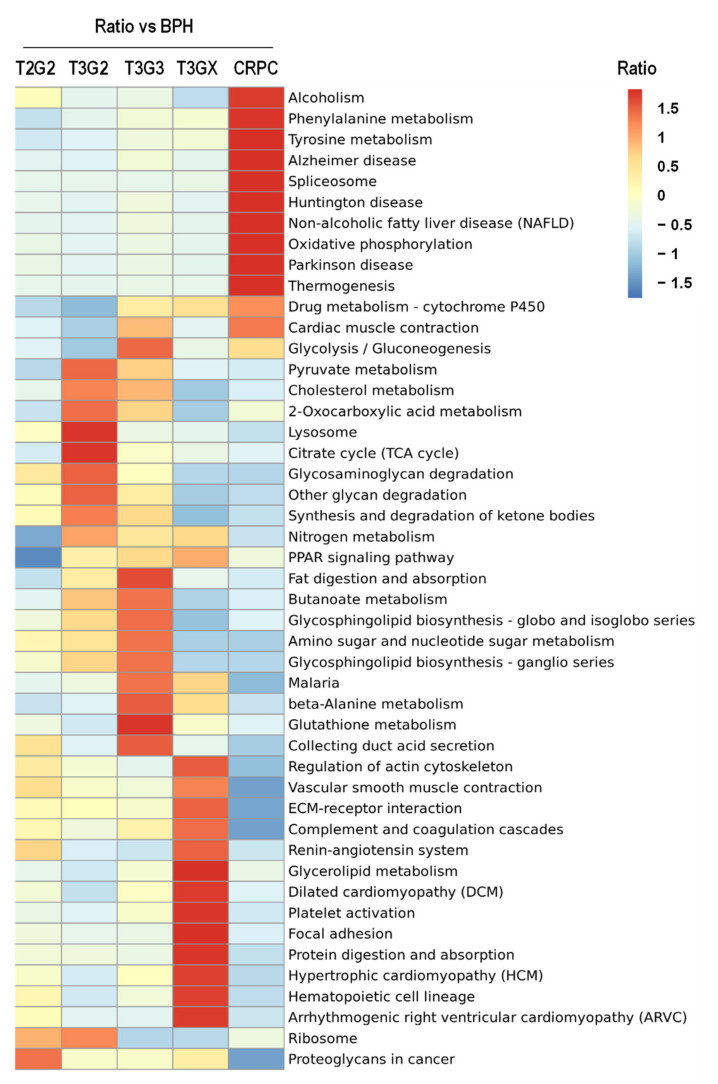
A comprehensive heatmap for cluster analysis of the enrichment patterns for the Kyoto Encyclopedia of Genes and Genomes pathway. The horizontal axis is the different protein grouping, and the vertical axis is the different functional classification. The red to blue gradient color represents the enrichment *p*-value (<0.05) of the protein grouping in the specified functional classification. Red represents significant enrichment, while blue represents no significant enrichment.

A hierarchical clustering analysis based on different functional classifications was performed to analyze the changes in protein levels in cancer tissues according to PCa progression stages and ADT in detail (Figure 2 and Figure S2). A heat map was displayed after one-way hierarchical clustering by transforming the enriched results with a *p*-value of <0.05 in each category. Overall, proteins were selectively enriched according to each PCa stage. Before (T3G3) and after (T3GX) ADT and CRPC, distinct tissue pathways were presented in the KEGG category. In T3G3 stage tissues, pathways related to glycosphingolipid metabolism and amino acid metabolism were enriched, whereas pathways related to cellular structures, such as actin and extracellular matrices, increased in T3GX. In CRPC, pathways related to phenylalanine and tyrosine metabolism increased. In addition to KEGG, it is possible to assess the selectively enriched protein information for each PCa progress stage in other GO category results, including GOBP, GOCC, GOMF, and the protein domain (Supplemental Figure S2). Based on this result, the protein expression significantly differed depending on the progression stage of PCa and the presence or absence of ADT.

### 3.3. Changes in Protein Expression Following ADT

**Figure 3:**
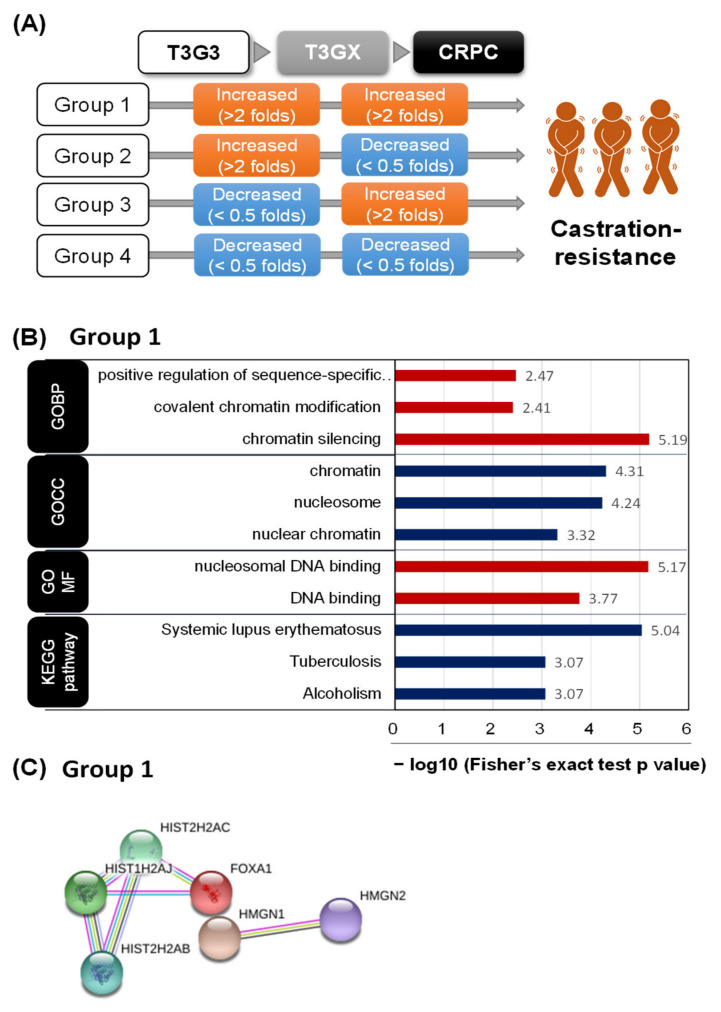
The expression of differential proteins based on cluster analysis among prostate cancer (PCa) stages. (**A**) Summary of cluster groups of differential proteins for visual exploration according to PCa stages. (**B**) Enriched gene ontology (GO) and Kyoto Encyclopedia of Genes and Genomes term: Group 1 represents increased proteins in both the T3GX/T3G3 and the CRPC/T3GX groups. The numbers of each bar reflect the proportion of differentially expressed proteins for every GO annotation. (**C**) STRING protein network for Group 1. CRPC, castration-resistant PCa; T3GX, patients with androgen deprivation therapy-treated T3G3 tissue.

To identify proteins that change before and after ADT, DEPs were specified among T3G3, T3GX, and CRPC. DEPs were defined as proteins with more than a two-fold change (|FC| ≥ 2) between stages. The changes in DEPs among T3G3, T3GX, and CRPC were systematically analyzed based on the amount of difference between tissues to examine the changes in protein levels in PCa tissues according to ADT in patients with PCa. The proteins analyzed in this process were categorized into four groups (Figure 3A). Proteins that doubled in T3GX compared with T3G3 and again doubled in CRPC compared with T3GX were classified as group 1 (Table 1). Group 2 contained proteins that doubled in T3GX but decreased in CRPC, and group 3, conversely, had proteins that increased after decreasing (Tables S2 and S3). Finally, group 4 contained the proteins that decreased from T3G3 to T3GX to CRPC.

The characteristics of each group were analyzed through a DAVID analysis (Figure 3B and Figure S3). In group 1, proteins (n = 15) related to chromatin and DNA binding were included in the GOBP, GOCC, and GOMF categories. Representative group 1 proteins included histone isoforms, hepatocyte nuclear factor 3-alpha (Forkhead box protein A1, FOXA1), and non-histone chromosomal protein HMG-14 (high mobility group nucleosome-binding domain-containing protein 1, HMGN 1). FOXA1 increased 3.3 times in T3GX and 22.4 times in CRPC compared with T3G3 and was the protein with the highest increase. The protein–protein network in group 1 is shown in Figure 3C, in which FOXA1 and histone 2A were linked. According to the DAVID analysis, proteins from group 2 were annotated in muscle contraction in GOBP, myosin and troponin complex in GOCC, cardiac muscle contraction, and cardiomyocytes in the KEGG category (Figure S3A). According to individual DEPs, 10 out of 41 group 2 proteins were myosin- and troponin-related proteins (Table S2). This result suggests that such proteins cause structural changes in the cells as they progress to ADT and CRPC. Although not shown in the DAVID analysis, 12 immunoglobulin proteins were included in group 2, where the distribution of the immune system in the PCa tissue could be changed during the process with ADT and CRPC.

Group 3 was annotated in positive regulation of the cell growth and oxidation-reduction process in GOBP, oxidoreductase activity in GOMF, and metabolic pathway in KEGG (Figure S3B). It was suggested that PCa cells might be weakened owing to a decrease in the intracellular metabolic system by ADT. Group 4 contained only four proteins, and the DAVID analysis could not be performed owing to the small number of proteins (Table 2). Of these four proteins, testis-expressed basic protein 1 (TSBP1) decreased the most, with reductions of 70% in T3GX compared with T3G3 and 92% in CRPC compared with T3GX.

### 3.4. Verification of Differentially Expressed Proteins in Clinical Tissues

**Figure 4:**
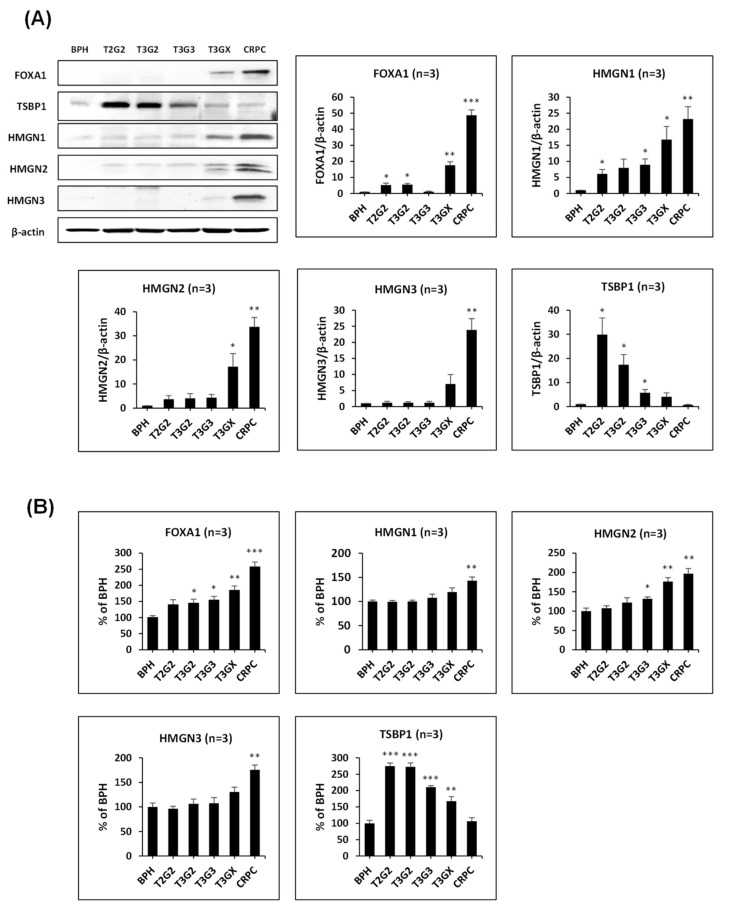
Validation of STRING predicted functions according to prostate cancer (PCa) stages. (**A**) Immunoblotting and relative ratio of protein level (n = 3). (**B**) Relative ratio of different stages of PCa patient tissue compared with the benign prostatic hyperplasia group using enzyme-linked immunosorbent assay (n = 3). * *p* < 0.05, ** *p* < 0.01, *** *p* < 0.001 vs. BPH.

We performed the immunoblot assay to confirm the quantitative change indicated by proteomics analysis (Figure 4A and Figure S4A) and verify the DEPs of comparative proteome results. Five proteins were selected for verification: four of them belonging to group 1 and one to group 4. In group 1, FOXA1 and HMGN1–3 were selected, and in group 4, TSBP1 was selected, which showed the highest decrease. The expression of FOXA1 and HMGN1 was verified in three additional tissues by immunoblot (Figure S4B). The immunoblot assay was performed for each patient using the same sample from the proteomic analysis, and quantitative values were displayed among the patient samples. Overall, it was confirmed that the immunoblot results correlated with the quantified value of the proteomic analysis. For FOXA1, a decrease was observed in T3G3 in the immunoblot results, whereas there was no difference compared with T3G2 in the proteome results. Additionally, it was verified that it significantly increased in T3GX and CRPC. It was confirmed that the HMGN family also continued to increase with PCa progression from T2G2 to CRPC. In the case of TSBP1, it was found that the group 4 protein decreased as it progressed to CRPC.

We performed the indirect enzyme-linked immunosorbent assay (ELISA) using prostate tissue from BPH to CRPC (Figure 4B) to further confirm the immunoblot results. The number of patients used for each stage is shown in Figure 4B. The reading ratios are shown in Table S4. The related protein levels of FOXA1 and HMGN1–3 significantly increased in CRPC tissue, as expected. TSBP1 significantly increased in T2G2 and T3G2, decreasing to the same level as BPH as it progressed to CRPC. Subsequently, the quantification of patient tissues was evaluated based on the selective antibodies of five proteins to verify the proteome-based quantitative results.

## 4. Discussion

In the therapy of patients with PCa, the progression of cancer cells to CRPC is the most fatal situation. Although the newly diagnosed patients are in the early stage, approximately 4% of them have metastatic cancer, and after local therapy, approximately 20–30% of them relapse and require systemic therapies [14]. ADT causes a temporary reduction in PCa tumor burden, but the malignancy begins to grow again, despite the lack of testicular androgens to form CRPC [15]. Patients with CRPC showed an increase in PSA levels or worsening of clinical findings despite maintaining the blood testosterone concentration at the castration level (<50 ng/dL) through ADT [16]. Even with successful ADT, tumors exhibit castration resistance because they increase the expression of intracellular steroid synthase genes so that testosterone and dihydrotestosterone (DHT) can be produced with only a trace amount of intracellular androgen precursors [17]. Moreover, despite the concentration of androgen maintained at the level of castration, cancer cells increase with the continuous transcriptional activity of the AR.

We quantified more than 4069 proteins between CRPC and PCa tissues before and after ADT based on a quantitative proteomic technology and identified new factors involved in the progression to CRPC. We analyzed DEP distribution among T3G3, T3GX, and CRPC and noted 15 proteins (group 1) with increased expression levels despite ADT. The expression levels of these proteins increased without responding to ADT and are expected to have the highest correlation with the castration restriction of PCa. Among the 15 and all the other quantified proteins, FOXA1 was the protein that increased most rapidly in CRPC. FOXA1, a subtype of hepatocyte nuclear factor superfamily, is a transcriptional regulator representing a unique subclass of DNA binding protein [18]. Previous studies already associated FOXA1 with PCa. A 2005 study, which used LPR-Tag LADY transgenic PCa mice models, showed that FOXA1 was differentially expressed in prostate diseases, with upregulation in both pre-neoplastic lesions and adenocarcinomas [19]. In FOXA1, protein expression was consistently observed in human PCa and expressed in PCa lines.

AR and androgen are essential during the progression to CRPC. Specifically, AR reactivation plays a pivotal role in the proliferation and survival of normal PCa cells to castration resistance. FOXA1 functions as a pioneer factor facilitating AR transcription and PCa growth [20]. It is known that FOXA1 modulates AR activity in metastatic PCa and that high-level nuclear FOXA1 expression is detected in 19% of primary and 89% of metastatic prostate tumors [20]. The increased FOXA1 expression causes the overactive AR complex to respond to low DHT levels [21]. Although there is FOXA1-independent AR signaling, the high levels of FOXA1 are still closely related to the progression to metastatic PCa. The novelty of our study was the discovery that FOXA1 expression increased even with ADT, but it increased explosively during CRPC. The mechanism through which FOXA1 increased during ADT remains unknown. Nonetheless, if it occurs as a reward mechanism for ADT, a new treatment capable of suppressing FOXA1 expression during ADT should be considered and combined with existing ones.

We verified that the HMGN1–3 expression among proteins belonging to group 1 increased in PCa tissue after ADT (T3GX) and in CRPC using immunoblot and indirect ELISA (Figure 4). HMGN is a family of non-histone chromatin architectural proteins ubiquitously expressed in the nuclei of vertebrates. Proteins from this family bind to the chromatin regulatory site, including enhancers and promoters [22]. In addition to the physiological functions of HMGN in diverse diseases, several studies reported the role of HMGN in cancer. HMGN1 expression was significant in highly metastatic MDA-MB-435 cells from breast cancer cell lines [23]. High cytoplasmic HMGN1 expression was associated with prominent histological grade and levels of tumor-infiltering lymphocytes in HER2-positive breast cancer tissues [24]. The HMGN2 expression level in human leukemia cells from patients with chronic myelogenic leukemia was three times higher than normal leukocytes [25]. To date, only a handful of studies reported the association between HMGN3 and cancer. Although it was not detected in this study, HMGN5 is overexpressed in PCa cells and plays an oncogenic role in PCa tumorigenesis and development while activating the mitogen-activated protein kinase signaling pathway [26]. Studies on the progression and association of HMGN1–3 and PCa still need to be developed, but considering the protumor effect of the HMGN family, we believe it may also be involved in PCa progression. In our study, HMGN2 increased twice compared with BPH in CRPC and HMGN1 and HMGN3 also increased from T2G2 to CRPC. Although HMGN1–3 did not cluster with FOXA1 in the PPI analysis, it may be related to FOXA1, which regulates transcription in the CRPC process as a histone binding protein.

## 5. Conclusions

In conclusion, based on comparative proteome analysis, we quantified 4069 proteins in the PCa tissues, including T2G2, T3G2, T3G3, T3GX, and CRPC, to find novel regulating factors associated with the progression to CRPC. DEPs were identified, including FOXA1 and HMGN1–3, the expression of which is increased during ADT process, to determine the regulator of progression to CRPC after ADT in clinical tissues. The role of the identified factors in the low stage of PCa should be elucidated through further studies, and their value as new treatment targets should be further evaluated.

## Figures and Tables

**Table 1:** Protein list of group 1, indicating a continuous increase from T3G3 to CRPC patients.

Protein Accession	Protein Description	Gene Name	Relative Ratio		
			T3GX/ T3G3	CRPC/ T3GX	CRPC/ T3G3
Q99878	Histone H2A type 1-J	*HIST1H2AJ*	3.9	2.3	8.8
Q15651	High mobility group nucleosome-binding domain-containing protein 3	*HMGN3*	3.5	3.9	13.4
Q8IUE6	Histone H2A type 2-B	*HIST2H2AB*	3.4	2.1	7.1
P55317	Hepatocyte nuclear factor 3-alpha	*FOXA1*	3.2	22.4	72.8
Q9NZN5	Rho guanine nucleotide exchange factor 12	*ARHGEF12*	3.1	3.7	11.9
P05204	Non-histone chromosomal protein HMG-17	*HMGN2*	3.1	4.8	14.6
Q14197	Peptidyl-tRNA hydrolase ICT1, mitochondrial	*MRPL58*	3.0	6.0	17.9
Q16777	Histone H2A type 2-C	*HIST2H2AC*	2.9	2.0	5.8
Q30134	HLA class II histocompatibility antigen, DRB1-8 beta chain	*HLA-DRB1*	2.7	3.2	8.7
P33241	Lymphocyte-specific protein 1	*LSP1*	2.4	2.1	5.0
P05114	Non-histone chromosomal protein HMG-14	*HMGN1*	2.4	2.7	6.5
Q8WU39	Marginal zone B- and B1-cell-specific protein	*MZB1*	2.2	2.1	4.5
Q6RW13	Type-1 angiotensin II receptor-associated protein	*AGTRAP*	2.1	4.0	8.5
P12107	Collagen alpha-1(XI) chain	*COL11A1*	2.0	4.2	8.4
Q9H7N4	Splicing factor, arginine/serine-rich 19	*SCAF1*	2.0	3.7	7.3

**Table 2:** Protein list of group 4, indicating a continuous decrease from T3G3 to CRPC patients.

Protein Accession	Protein Description	Gene Name	Relative Ratio		
			T3GX/ T3G3	CRPC/ T3GX	CRPC/ T3G3
Q5SRN2	Testis-expressed basic protein 1	*TSBP1*	0.30	0.08	0.02
P02788	Lactotransferrin	*LTF*	0.31	0.49	0.15
P38571	Lysosomal acid lipase/cholesteryl ester hydrolase	*LIPA*	0.47	0.44	0.21
P33947	ER lumen protein-retaining receptor 2	*KDELR2*	0.47	0.35	0.17

CRPC, castration-resistant prostate cancer; T3GX, patients with androgen deprivation therapy-treated T3G3 tissue.

## Data Availability

The data presented in this study are available on request from the corresponding author. The mass spectrometry proteomics data have been deposited into the ProteomeXchange Consortium via the PRIDE partner repository with the dataset identifier PXD023592.

## Supplementary Materials

The following data are available online at https://www.mdpi.com/article/10.3390/cancers13143432/s1. Table S1: Summary of differentially expressed proteins; Table S2: Protein list of group 2; Table S3: Protein list of group 3; Table S4: Densitometry readings ratio of each band (A), ELISA reading ratio of 5 proteins (B); Figure S1: Heatmap of Pearson correlation coefficients from all quantified proteins between each pair of samples; Figure S2: A comprehensive heatmap for cluster analysis of the enrichment patterns. (A) Biological process, (B) Cellular component, (C) Molecular function, (D) Protein domain; Figure S3: Bioinformatics analysis of differentially expressed proteins in T3G3/T3GX/CRPC. (A) Gene ontology and Kyoto Encyclopedia of Genes and Genomes annotation of the proteins increased in the T3GX/T3GX group but decreased in the CRPC/T3G3 group. (B) Protein interactions by STRING network according to group 2 of cluster analysis. (C) Gene ontology and Kyoto Encyclopedia of Genes and Genomes annotation of differentially expressed proteins decreased in the T3GX/T3GX group but increased in the CRPC/T3G3 group; Figure S4. The Whole blot of five proteins.
