# Supplementary material for: Characterization of Novel Progression Factors in Castration-Resistant Prostate Cancer Based on Global Comparative Proteome Analysis
Source: Cancers (Basel). 2021 Jul 8;13(14):3432. doi: 10.3390/cancers13143432 (PMC8304965; doi:10.3390/cancers13143432)
Supplement: Supplementary file 1 [file cancers-13-03432-s001.zip › cancers-1216170-supplementary for xml.pdf]

cancers

MDPI

Characterization of Novel Progression Factors in Castration-Resistant Prostate Cancer Based on Global Comparative Proteome Analysis

Ann-Yae Na, Soyoung Choi, Eunju Yang, Kwang-Hyeon Liu, Sunghwan Kim, Hyun Jin Jung, Youngshik Choe, Yun-Sok Ha, Tae Gyun Kwon, Jun Nyung Lee and Sangkyu Lee

Legends of Supplementary Tables and Figures

Table S1. Summary of differentially expressed proteins

Table S2. Protein list of Group 2

Table S3. Protein list of Group 3

Table S4. Densitometry readings ratio of each band (A), ELISA reading ratio of 5 proteins (B)

Figure S1. Heatmap of Pearson correlation coefficients from all quantified proteins between each pair of samples

Figure S2. A comprehensive heatmap for cluster analysis of the enrichment patterns; (A) Biological process, (B) Cellular component, (C) Molecular function, (D) Protein domain.

Figure S3. Bioinformatics analysis of differential expressed proteins in T3G3/T3GX/CRPC. (A) GO and KEGG annotation of that increased in T3GX/T3GX group but decreased CRPC/T3G3 group. (B) Protein interactions by STRING network according to group 2 of cluster analysis. (C) GO and KEGG annotation of differential expressed proteins that decreased in T3GX/T3GX group, but increased CRPC/T3G3 group.

Figure S4. The Whole blot of five proteins

Table S2. Protein list of Group 2.

Protein accession	*Protein description*	*MW [kDa]*	*Gene name*	T3GX/ T3G3	CRPC/ T3GX	CRPC/ T3G3
P10916	Myosin regulatory light chain 2, ventricular/cardiac muscle isoform	18.79	MYL2	7.73	0.15	1.18
P07098	Gastric triacylglycerol lipase	45.24	LIPF	6.98	0.15	1.08
P63316	Troponin C, slow skeletal and cardiac muscles	18.40	TNNC1	6.29	0.13	0.80
P45378	Troponin T, fast skeletal muscle	31.82	TNNT3	5.99	0.23	1.38
A0A0B4J1X5	Immunoglobulin heavy variable 3-74	12.84	IGHV3-74	5.61	0.10	0.57
A0A075B6K4	Immunoglobulin lambda variable 3-10	12.44	IGLV3-10	5.58	0.24	1.34
P13805	Troponin T, slow skeletal muscle	32.95	TNNT1	4.86	0.20	0.97
P07451	Carbonic anhydrase 3	29.56	CA3	4.80	0.15	0.74
P01709	Immunoglobulin lambda variable 2-8	12.38	IGLV2-8	4.41	0.36	1.58
P01591	Immunoglobulin J chain	18.10	JCHAIN	4.36	0.50	2.18
Q96A32	Myosin regulatory light chain 2, skeletal muscle isoform	19.01	MYLPF	4.23	0.29	1.22
P11678	Eosinophil peroxidase	81.04	EPX	4.18	0.41	1.70
Q5VXH5	PRAME family member 7	53.63	PRAMEF7	4.04	0.32	1.28
P14649	Myosin light chain 6B	22.76	MYL6B	3.98	0.35	1.38
P30711	Glutathione S-transferase theta-1	27.34	GSTT1	3.96	0.34	1.34
Q05315	Galectin-10	16.45	CLC	3.92	0.26	1.00
P19237	Troponin I, slow skeletal muscle	21.69	TNNI1	3.87	0.29	1.11
P05976	Myosin light chain 1/3, skeletal muscle isoform	21.15	MYL1	3.81	0.47	1.77
P02144	Myoglobin	17.18	MB	3.73	0.19	0.71
P08590	Myosin light chain 3	21.93	MYL3	3.60	0.46	1.67
P12724	Eosinophil cationic protein	18.39	RNASE3	3.44	0.28	0.96
P01714	Immunoglobulin lambda variable 3-19	12.04	IGLV3-19	3.30	0.41	1.35
P0DOX8	Immunoglobulin lambda-1 light chain	22.83	---	3.01	0.46	1.40
P0DOX5	Immunoglobulin gamma-1 heavy chain	49.33	---	2.96	0.27	0.81
P13929	Beta-enolase	46.99	ENO3	2.95	0.31	0.91
A2RTY3	Protein HEATR9	65.68	HEATR9	2.94	0.20	0.58
P12883	Myosin-7	223.09	MYH7	2.91	0.39	1.13
P01833	Polymeric immunoglobulin receptor	83.28	PIGR	2.89	0.40	1.16
P01834	Immunoglobulin kappa constant	11.77	IGKC	2.85	0.44	1.24
A0A075B6I9	Immunoglobulin lambda variable 7-46	12.47	IGLV7-46	2.70	0.44	1.19
A0A0B4J2H0	Immunoglobulin heavy variable 1-69D	12.66	IGHV1-69D	2.54	0.37	0.95

A0A075B6H9	Immunoglobulin lambda variable 4-69	12.77	IGLV4-69	2.46	0.40	0.98
Q0D2J5	Zinc finger protein 763	46.10	ZNF763	2.46	0.19	0.47
P09488	Glutathione S-transferase Mu 1	25.71	GSTM1	2.36	0.15	0.35
P25189	Myelin protein P0	27.55	MPZ	2.28	0.50	1.13
P02743	Serum amyloid P-component	25.39	APCS	2.25	0.45	1.01
P60903	Protein S100-A10	11.20	S100A10	2.24	0.33	0.74
P17540	Creatine kinase S-type, mitochondrial	47.50	CKMT2	2.20	0.45	1.00
P59666	Neutrophil defensin 3	10.25	DEFA3	2.15	0.47	1.01
Q9BVA1	Tubulin beta-2B chain	49.95	TUBB2B	2.02	0.30	0.61
A0A0C4DH31	Immunoglobulin heavy variable 1-18	12.82	IGHV1-18	2.00	0.27	0.54

Table S3. Protein list of Group 3.

Protein accession	*Protein description*	*MW [kDa]*	*Gene name*	*T3GX /T3G3*	*CRPC /T3GX*	*CRPC /T3G3*
Q00796	Sorbitol dehydrogenase	38.32	SORD	0.30	3.02	0.91
P16870	Carboxypeptidase E	53.15	CPE	0.32	2.54	0.80
Q29836	HLA class I histocompatibility antigen, B-67 alpha chain	40.34	HLA-B	0.35	9.02	3.17
P14555	Phospholipase A2, membrane associated	16.08	PLA2G2A	0.41	2.53	1.04
P80723	Brain acid soluble protein 1	22.69	BASP1	0.42	7.95	3.35
Q7Z5M5	Transmembrane channel-like protein 3	125.68	TMC3	0.43	3.94	1.68
O76054	SEC14-like protein 2	46.15	SEC14L2	0.45	2.40	1.09
Q6ZSS7	Major facilitator superfamily domain-containing protein 6	88.09	MFSD6	0.46	3.33	1.54
Q07092	Collagen alpha-1(XVI) chain	157.75	COL16A1	0.47	2.09	0.98
Q53GD3	Choline transporter-like protein 4	79.25	SLC44A4	0.49	3.09	1.51
P28907	ADP-ribosyl cyclase/cyclic ADP-ribose hydrolase 1	34.33	CD38	0.49	2.43	1.19
Q9Y394	Dehydrogenase/reductase SDR family member 7	38.30	DHRS7	0.49	2.46	1.21
P49327	Fatty acid synthase	273.42	FASN	0.50	2.68	1.35
Q9ULV0	Unconventional myosin-Vb	213.67	MYO5B	0.50	2.04	1.03

Table S4A. Densitometry readings ratio of each band.

**Proteins**	**Normalized ratio (vs β-actin) (n=3)**											
	**BPH**		**T2G2**		**T3G2**		**T3G3**		**T3GX**		**CRPC**	
	**AVE**	**SE**	**AVE**	**SE**	**AVE**	**SE**	**AVE**	**SE**	**AVE**	**SE**	**AVE**	**SE**
FOXA1	1.00	0.00	5.21	1.29	5.46	0.76	1.10	0.33	17.57	2.21	48.71	3.42
TSBP1	1.00	0.00	29.93	6.89	17.36	4.26	5.78	1.33	4.08	1.59	0.67	0.24
HMGN1	1.00	0.00	6.09	1.43	7.98	2.75	8.93	1.82	16.80	4.08	23.18	3.86
HMGN2	1.00	0.00	3.67	1.46	4.06	1.94	4.30	1.38	17.18	5.42	33.70	3.93
HMGN3	1.00	0.00	1.19	0.46	1.21	0.29	1.22	0.41	7.04	2.91	23.85	3.53

Table S4B. ELISA reading ratio of 5 proteins.

**Proteins**	**Relative % of BPH (n=3)**											
	**BPH**		**T2G2**		**T3G2**		**T3G3**		**T3GX**		**CRPC**	
	**AVE**	**SE**	**AVE**	**SE**	**AVE**	**SE**	**AVE**	**SE**	**AVE**	**SE**	**AVE**	**SE**
FOXA1	100.00	5.70	139.39	15.76	144.21	13.15	153.95	12.02	184.39	13.19	256.84	15.41
TSBP1	100.00	9.04	274.70	9.62	272.27	12.62	210.24	4.77	167.55	14.29	106.31	10.99
HMGN1	100.00	3.12	99.57	2.33	100.08	2.69	107.70	7.73	119.70	8.55	143.06	7.69
HMGN2	100.00	7.96	107.34	6.51	122.13	12.60	131.63	4.89	176.47	10.41	196.52	13.74
HMGN3	100.00	8.08	96.22	5.02	106.33	9.90	107.50	11.62	130.42	9.88	175.78	9.66

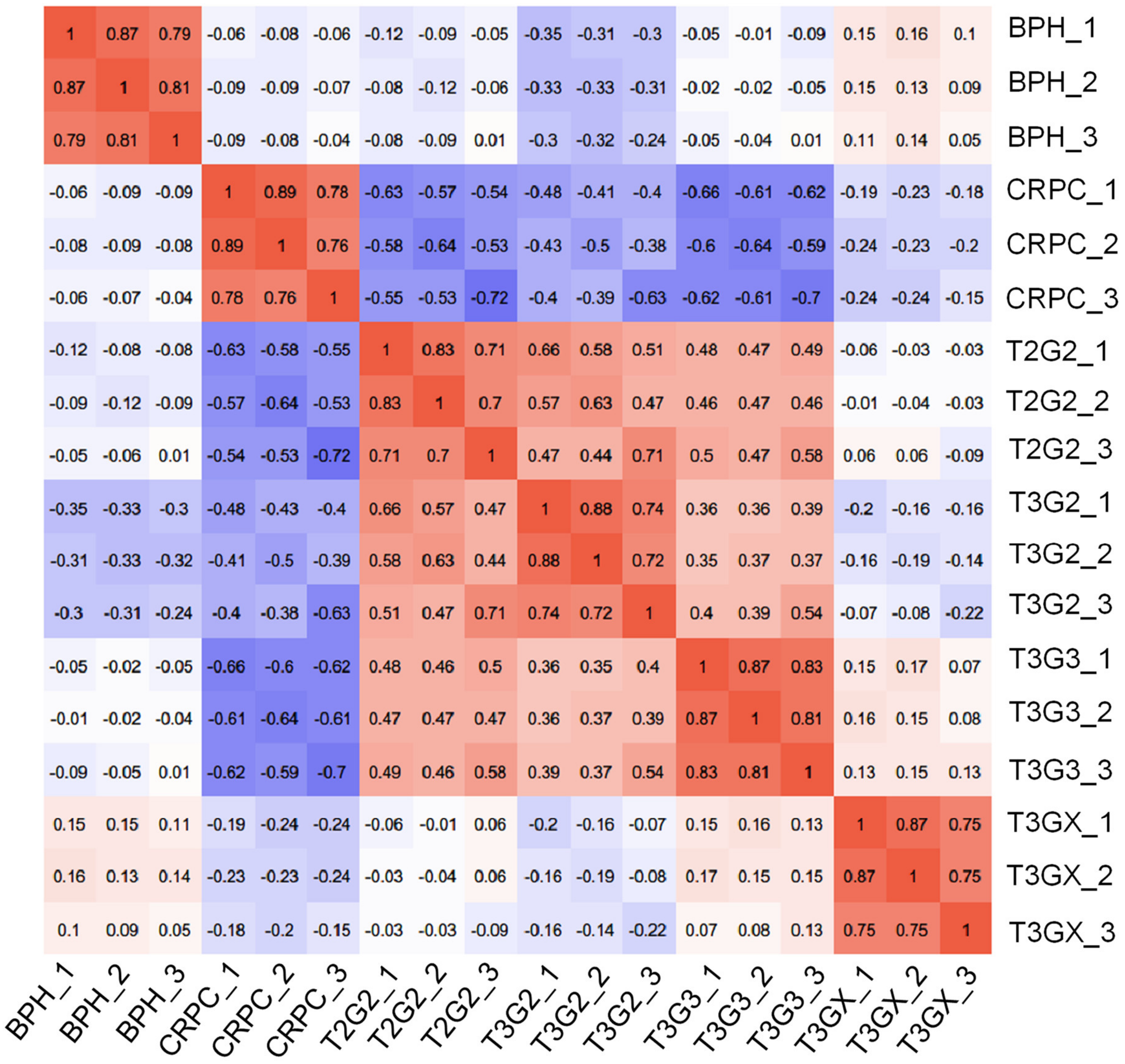

Figure S1. Heatmap of Pearson correlation coefficients from all quantified proteins between each pair of samples.

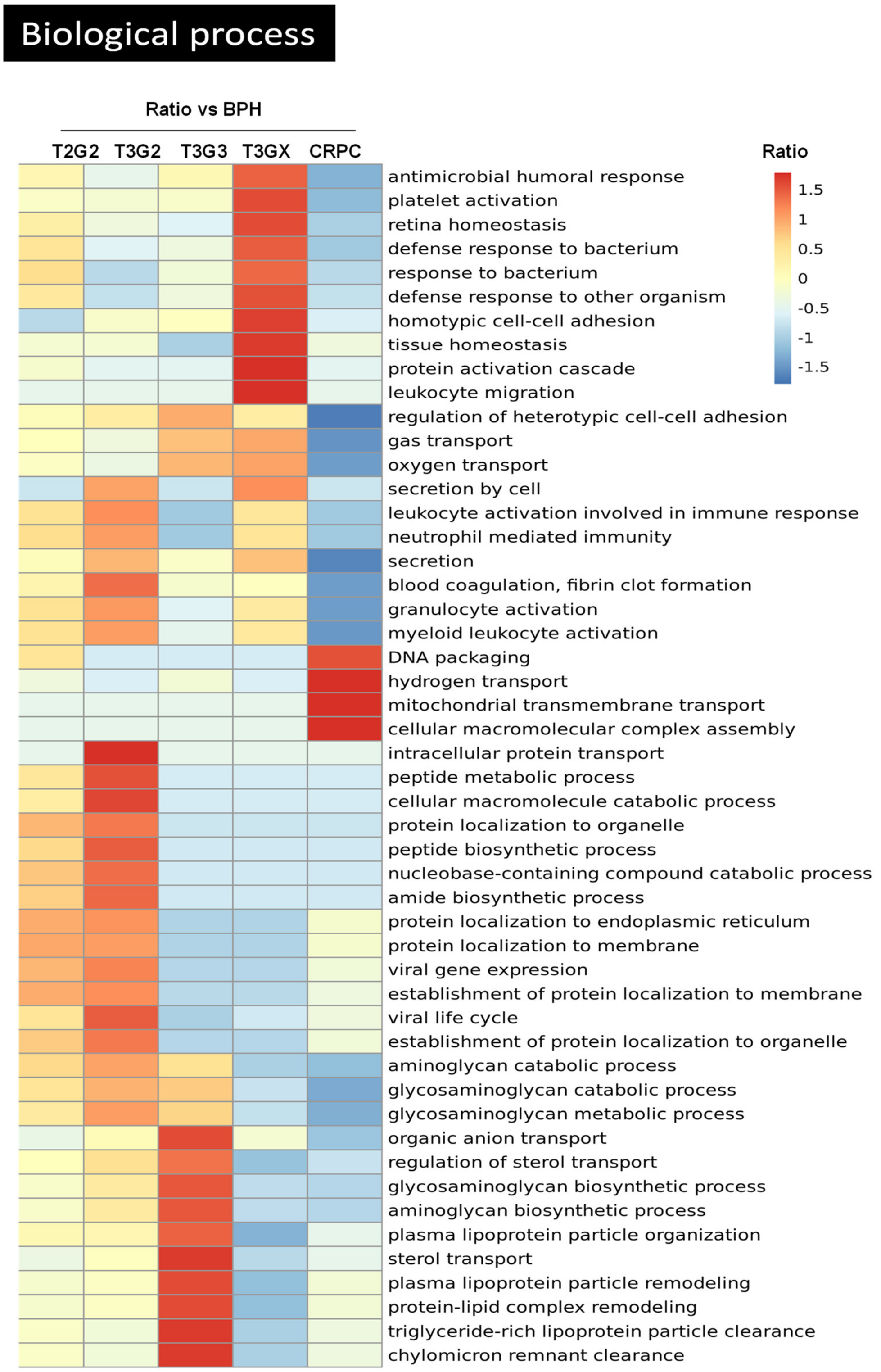

Figure S2. A comprehensive heatmap for cluster analysis of the enrichment patterns; (A) Biological process, (B) Cellular component, (C) Molecular function, (D) Protein domain (Continued).

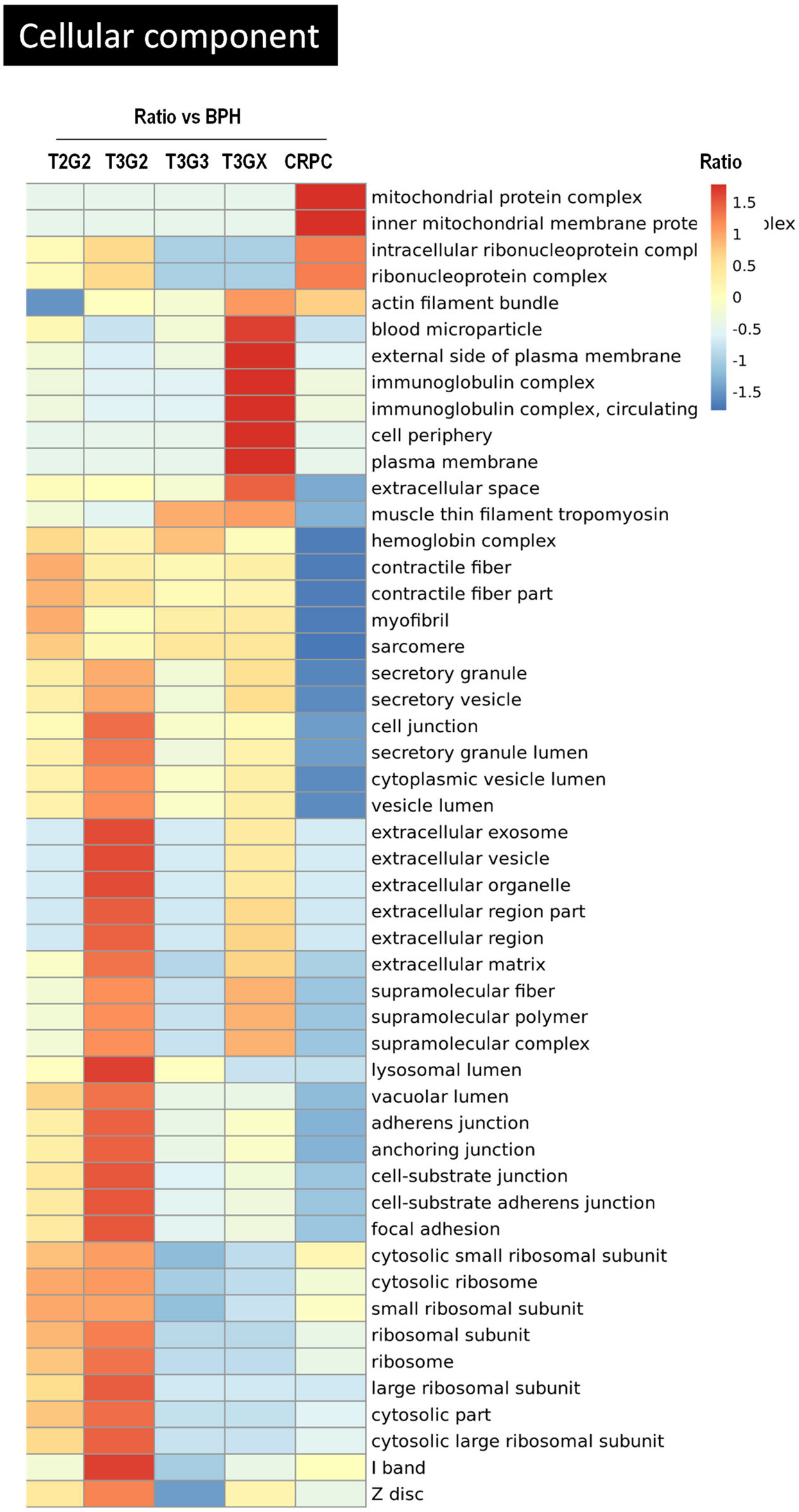

Figure S2. A comprehensive heatmap for cluster analysis of the enrichment patterns; (A) Biological process, (B) Cellular component, (C) Molecular function, (D) Protein domain (Continued).

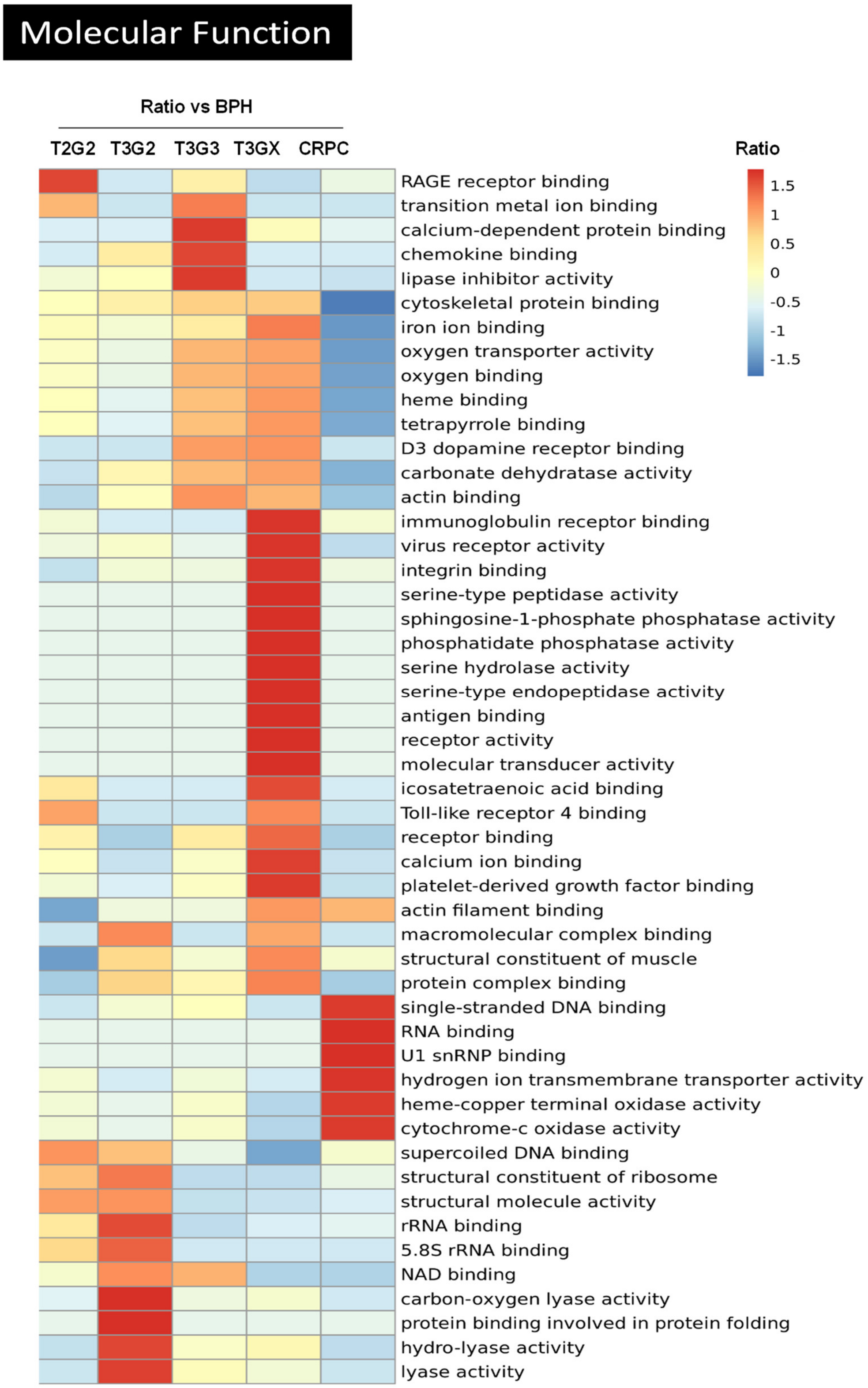

Figure S2. A comprehensive heatmap for cluster analysis of the enrichment patterns; (A) Biological process, (B) Cellular component, (C) Molecular function, (D) Protein domain (Continued).

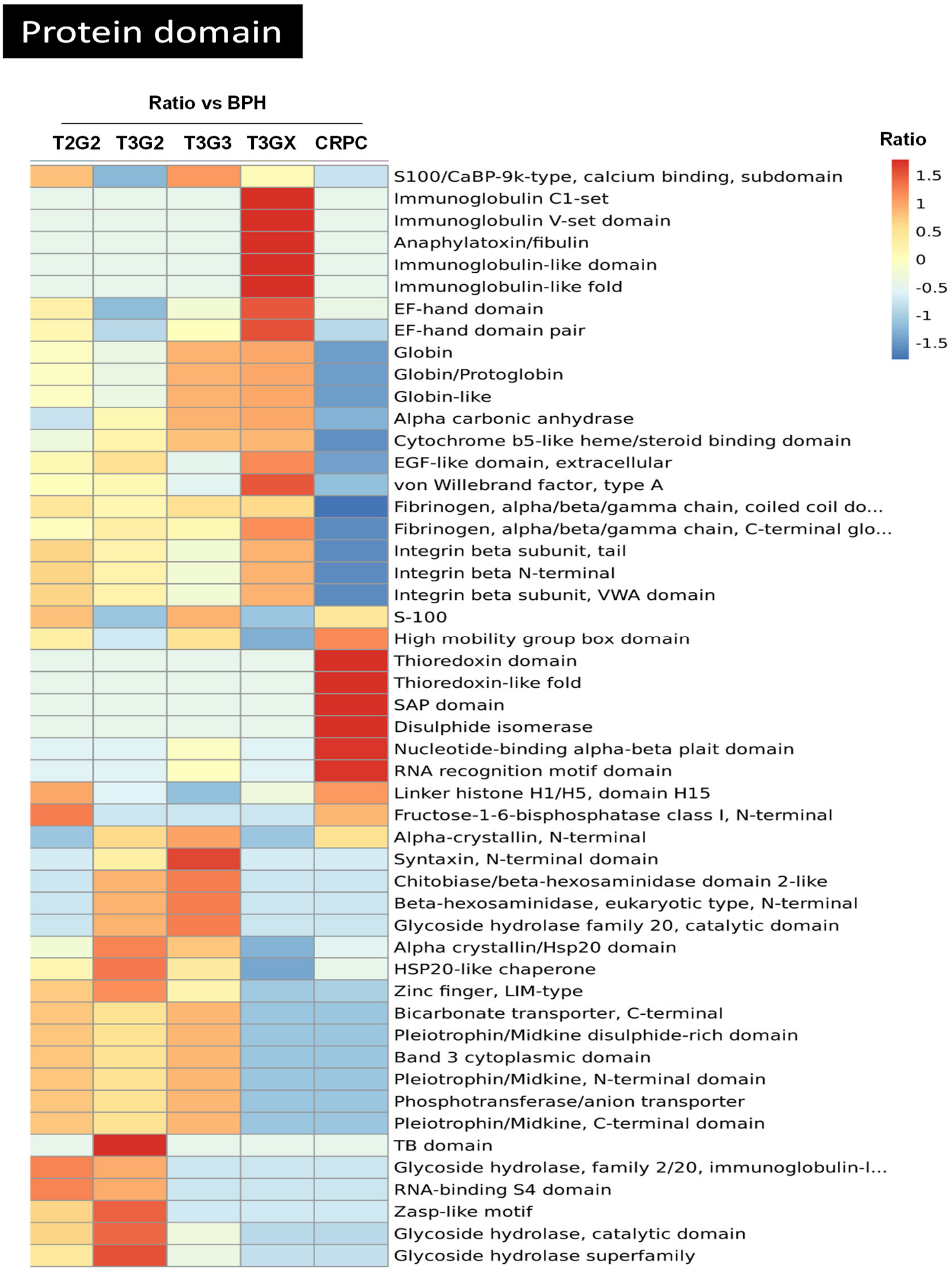

Figure S2. A comprehensive heatmap for cluster analysis of the enrichment patterns; (A) Biological process, (B) Cellular component, (C) Molecular function, (D) Protein domain.

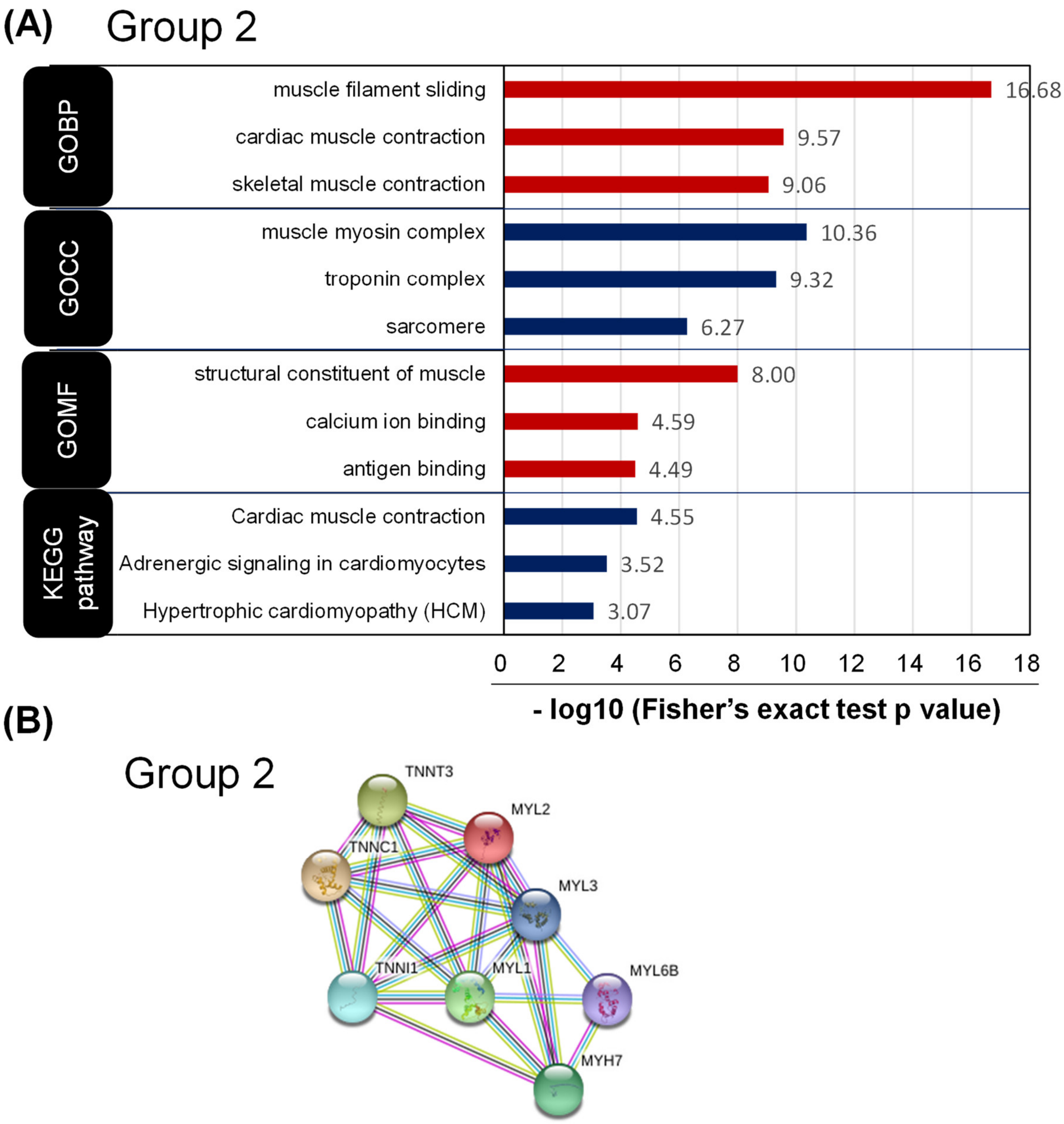

Figure S3. Bioinformatics analysis of differential expressed proteins in T3G3/T3GX/CRPC. (A) GO and KEGG annotation of that increased in T3GX/T3GX group but decreased CRPC/T3G3 group. (B) Protein interactions by STRING network according to group 2 of cluster analysis. (C) GO and KEGG annotation of differential expressed proteins that decreased in T3GX/T3GX group, but increased CRPC/T3G3 group (Continued).

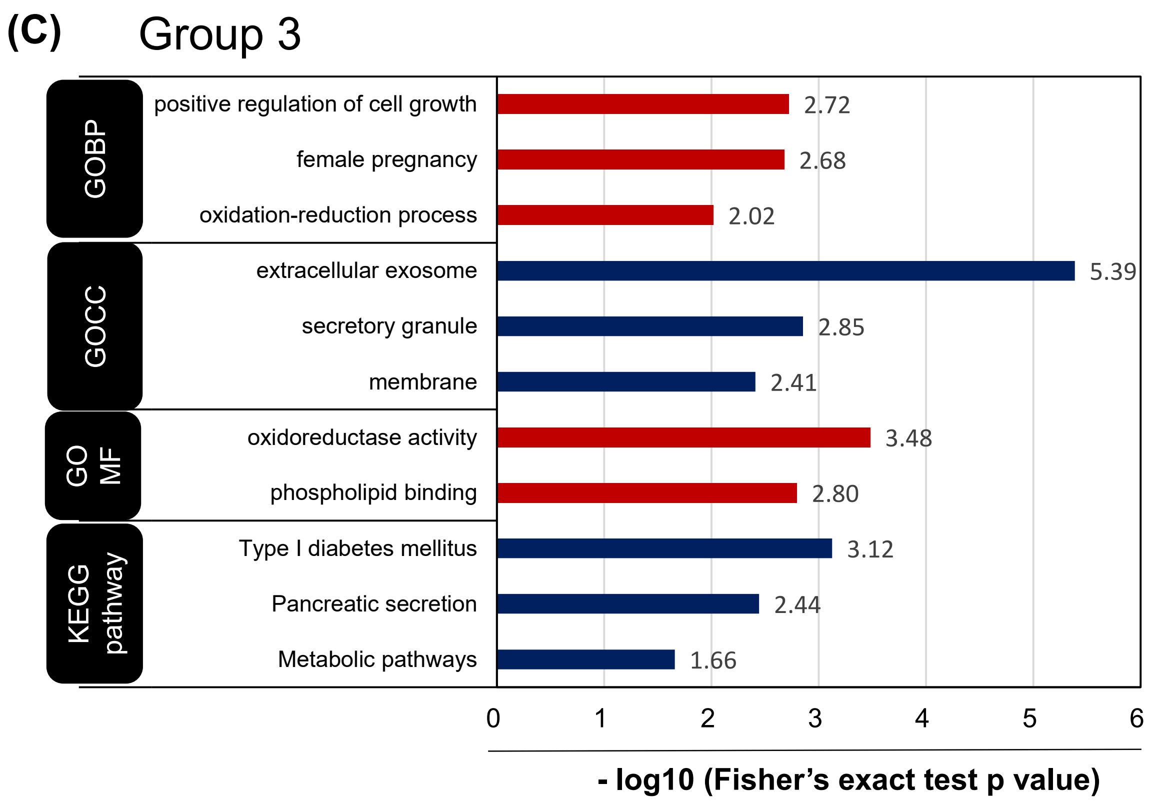

Figure S3. Bioinformatics analysis of differential expressed proteins in T3G3/T3GX/CRPC. (A) GO and KEGG annotation of that increased in T3GX/T3GX group but decreased CRPC/T3G3 group. (B) Protein interactions by STRING network according to group 2 of cluster analysis. (C) GO and KEGG annotation of differential expressed proteins that decreased in T3GX/T3GX group, but increased CRPC/T3G3 group.

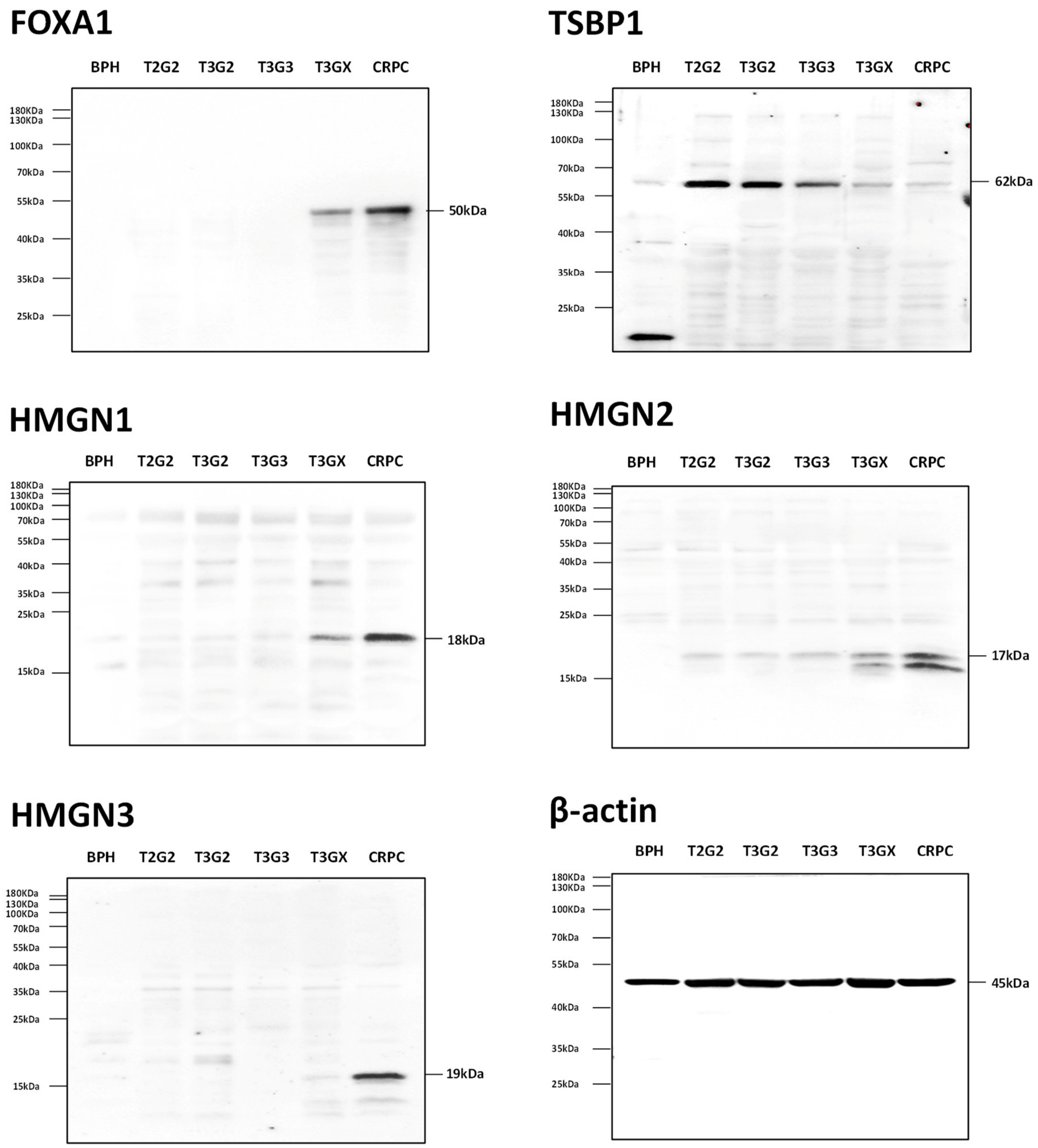

Figure S4A. The Whole blot of five proteins.

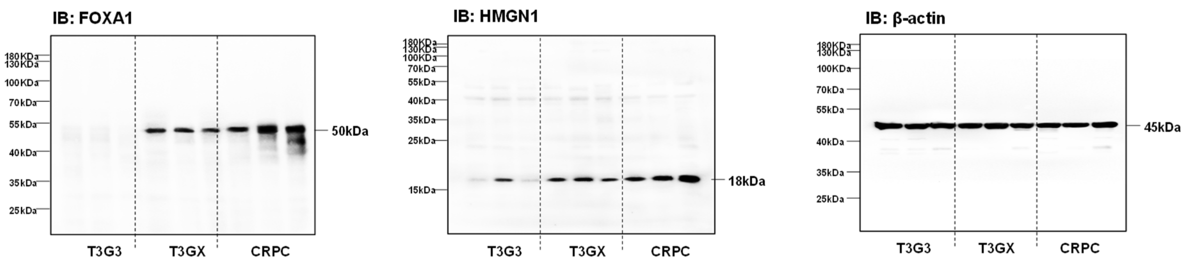

Figure S4B. The Individual whole blot of FOXA1 and HMGN1.
